# Case report: Spontaneous mandibular body regeneration following unilateral subtotal mandibulectomy in a 3-month-old French bulldog

**DOI:** 10.3389/fvets.2023.1281232

**Published:** 2023-10-11

**Authors:** Alexandra L. Wright, Santiago Peralta, Nadine Fiani

**Affiliations:** Department of Clinical Sciences, College of Veterinary Medicine, Cornell University, Ithaca, NY, United States

**Keywords:** spontaneous regeneration, mandibulectomy, squamous cell carcinoma, oral neoplasia, juvenile

## Abstract

**Objective:**

To document a case of spontaneous regeneration of the mandibular body following subtotal mandibulectomy in a juvenile dog.

**Case summary:**

A 3-month-old male intact French bulldog was presented with papillary oral squamous cell carcinoma located at the dorsal aspect of the molar region of the left mandible. Initial biopsy of the mass was performed by the primary care veterinarian. Complete clinical staging revealed no signs of metastasis. Computed tomographic images of the head showed minimal contrast enhancement of the mass with no signs of periosteal or bone involvement. Subtotal mandibulectomy was performed. Histopathology indicated complete excision of the tumor. The patient returned 8-weeks later for follow up and cleft palate surgical repair, at which time bone was noted in the mandibulectomy area on palpation. Repeat computed tomography of the head revealed complete regeneration of the left mandibular body from the level of the ramus to the mandibular symphysis. No treatment for malocclusion was necessary due to the reformation of a functional mandible.

**Clinical relevance:**

The present case demonstrates that spontaneous regeneration of the mandibular body is possible following subtotal mandibulectomy in immature dogs. Subtotal mandibulectomy is a radical procedure that can lead to long term complications including mandibular drift, malocclusion, and oral pain. This case report provides evidence that these sequelae may be mitigated or eliminated in young patients undergoing this procedure.

## Introduction

Squamous cell carcinoma is one of the most common malignancies of the oral cavity in dogs and several histological subtypes have been described (1, 2). The papillary subtype has previously been noted to occur in young dogs (less than 1 year old) (3–5), but it is also described to occur in adult dogs (2, 5). *En bloc* excision, when possible, is considered the gold standard treatment for canine papillary oral squamous cell carcinoma (pOSCC) (5). Depending on the location and extent of local invasion of the oral tumor, removal of extensive portions of the maxilla or mandible may be necessary to achieve clean margins. Excision of a considerable portion of the mandible results in changes to the stability and movement of the remaining mandibular anatomy, leading to mandibular drift, traumatic malocclusion, and changes to the temporomandibular joint (TMJ) (6–9). These sequelae can lead to chronic pain and alterations to prehension and mastication, potentially affecting the patient’s long-term quality of life (7).

In this case, the long-term consequences of a young puppy undergoing subtotal mandibulectomy were uncertain given the low prevalence of oral cancer in this age group. Other reports of pOSCC in young dogs only describe tumor recurrence rates in the follow-up period, but do not include clinical outcomes related to occlusion and mastication function (2–5). To the authors’ knowledge, this is the first report of spontaneous regeneration of the mandibular body following subtotal mandibulectomy in a juvenile dog, subsequently resulting in a functional oral occlusion postoperatively without further intervention.

## Case description

### Case presentation

**Figure 1:**
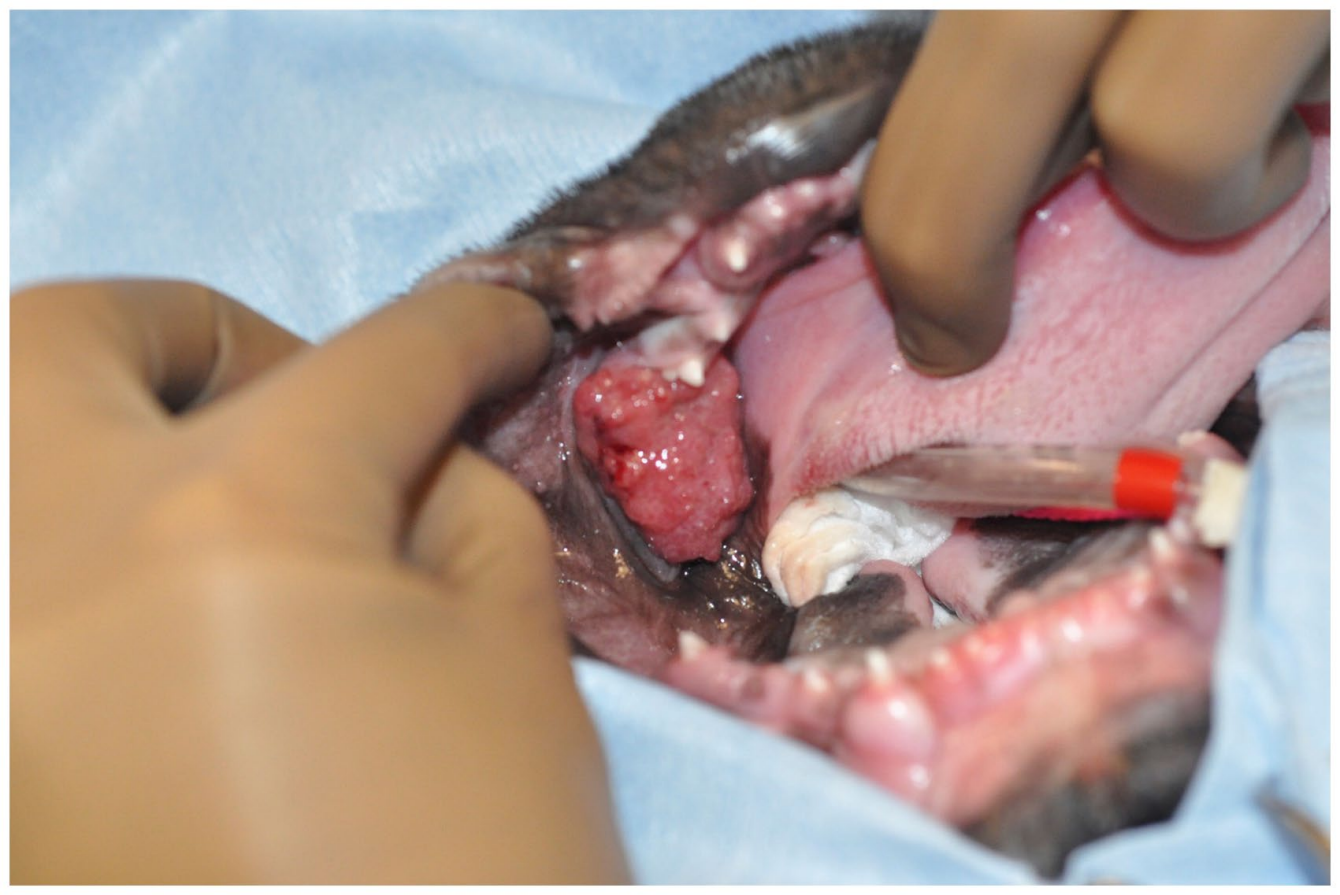
Image of the oral mass obtained intraoperatively. The patient is in dorsal recumbency, and the oral mass is located distal to the left mandibular deciduous third premolar tooth.

**Figure 2:**
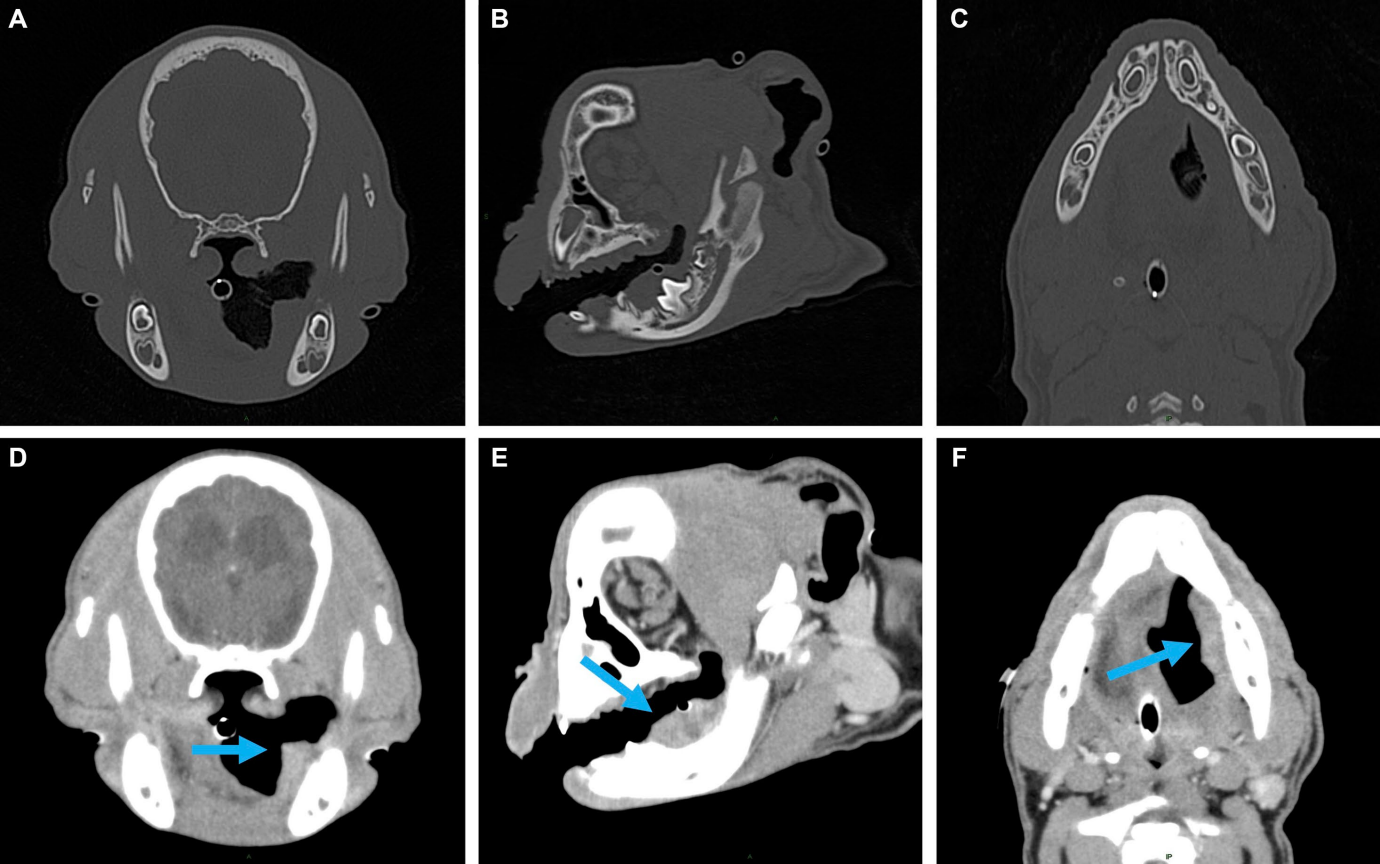
Representative multiplanar views of the surgical planning CT. **(A–C)** Transverse, sagittal and dorsal bone window views of the left caudal mandible. No evidence of lysis or periosteal reaction. **(D–F)** Transverse, sagittal and dorsal post-contrast enhancement soft tissue window views of soft tissue mass (indicated by the blue arrow). Adjacent to the developing left mandibular permanent first molar, a 2.5 × 1.6 × 1.1 cm, soft tissue attenuating, minimally contrast enhancing mass was noted. The mass extended rostrally to the level of the left mandibular deciduous third premolar and caudally along the medial margin of the left mandibular body.

A 3-month-old male intact French bulldog was presented for evaluation and treatment of a pOSCC located at the dorsal aspect of the caudal left mandibular body. The mass had been noted incidentally by the referring veterinarian 2 weeks prior while assessing a congenital palatal defect. Initial biopsy of the mass resulted in a diagnosis of a pOSCC. Routine bloodwork performed prior to referral revealed a mild regenerative anemia (HCT 34%) but was otherwise unremarkable. On presentation, a conscious oral examination revealed an ulcerated, broad-based soft tissue mass located distal to the left deciduous mandibular third premolar tooth (Figure 1). A midline defect of the soft and hard palate consistent with a complete bilateral cleft of the secondary palate was also identified (10). The remainder of the physical examination was normal. The following day, the patient returned for advanced imaging and staging of the mass. The patient was premedicated with dexmedetomidine (2 mcg/kg, IM) and methadone (0.2 mg/kg, IM), and general anesthesia was induced with propofol (9 mg/kg, IV). The dog was intubated, and general anesthesia was maintained with isoflurane and oxygen. Whole-body computed tomography (CT) was performed with and without contrast administration for surgical planning as well as tumor staging (Figure 2). The mass was minimally contrast enhancing and there were no signs of bone invasion. No signs of regional or distant metastasis were noted. Repeat incisional biopsy of the mass confirmed the initial diagnosis of pOSCC. Fine-needle aspirate of both mandibular lymph nodes showed no evidence of metastasis on the examined smears.

### Surgical treatment and outcome

The dog returned 2 days later for a left extended subtotal mandibulectomy to remove the mass. The extended subtotal mandibulectomy was elected with the goal of achieving clean margins while also preserving the anatomy of the ipsilateral TMJ (11). The same anesthetic protocol was utilized. Following intubation and placement of a pharyngeal pack, the oral cavity was rinsed with 0.12% chlorhexidine gluconate to minimize the bacterial burden. Supra- and subgingival ultrasonic scaling of all teeth was performed, leaving the affected mandible for last to avoid dissemination of neoplastic cells to other locations of the mouth. A left inferior alveolar nerve block was performed via an extraoral approach to avoid neoplastic tissue. The patient was placed in dorsal recumbency and surgically draped. An intraoral approach to the left mandible was performed. Soft tissue margins were planned 1 cm around the tumor with a surgical marker, and the incisions were made with a number 15 blade on the lingual and buccal aspects of the mandible starting at the level of the mandibular symphysis and extending caudally along the demarcated margins. The buccal and lingual mucosa was elevated from the mandible with a periosteal elevator. The mandibular symphysis was separated using an 8 mm osteotome and a 100 g mallet. Hemostasis was achieved with a combination of ligation or electrocautery when appropriate. The remaining soft tissue and muscular attachments of the mandible were elevated from the mandibular body. Lateral traction of the mandible provided visualization of the inferior alveolar neurovascular bundle. The vessels were ligated with 4–0 polyglactin 910 suture material prior to transection. Ostectomy 1 cm caudal to the mass was performed using a straight bone-cutting tip on a piezoelectric surgical handpiece (12). The surgical site was thoroughly lavaged with sterile 0.9% saline solution prior to routine apposition of the muscle and mucosal layers in a simple interrupted pattern using 5–0 poliglecaprone 25. The pharyngeal pack was removed, and the patient was positioned in right lateral recumbency for placement of an esophageal feeding tube.

The patient recovered from anesthesia and was hospitalized postoperatively on a constant rate infusion of fentanyl (3 μg/kg/h), carprofen (2.2 mg/kg, SQ, q12 h), and ampicillin/sulbactam (30 mg/kg, IV, q8 h). The patient tolerated the feeding tube well and appeared comfortable. The dog was discharged to the care of its owners the following day with carprofen (2.2 mg/kg, via feeding tube, q 12 h), gabapentin (10 mg/kg, via feeding tube, q8–12 h), and amoxicillin/clavulanic acid (13.75 mg/kg, via feeding tube, q12 h). The owners were instructed to feed the patient specific volumes through the feeding tube and prevent any access to chews or toys for 4 weeks.

**Figure 3:**
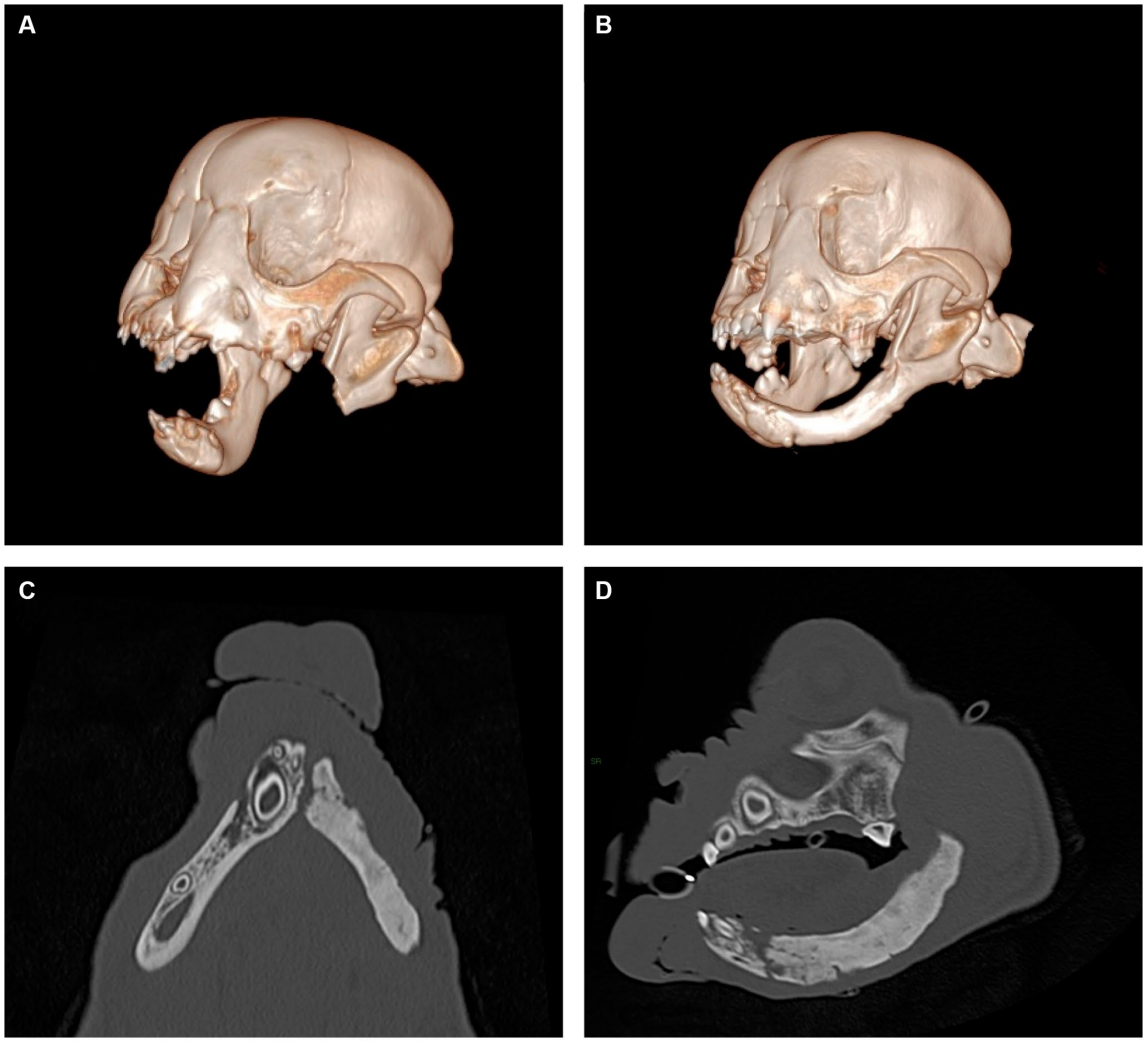
Representative views of computer-generated 3-D render of CT performed immediately post-operatively **(A)** and 8 weeks following surgery **(B)** and multiplanar views of newly formed mandible, dorsal view **(C)** and sagittal view **(D)**. New mandibular bone is noted to span the region from the osteotomy site to the mandibular symphysis. The appearance of the newly formed bone has internal trabecular markings that are sclerotic in appearance and a thin cortex present at the periphery. No overt mandibular canal is present.

Histopathology of the *en bloc* section of mandible reported complete surgical excision of the tumor. The patient returned 18 days after surgery, at which time a conscious oral exam revealed no evidence of mandibular drift or traumatic malocclusion. The patient returned again 8 weeks after the initial surgery for cleft palate repair, at which time firm tissue at the area of the mandibulectomy site was palpated on conscious oral examination. The same anesthetic protocol was utilized, and CT scan of the head was performed, revealing new mandibular bone formation from the ostectomy site to the level of the mandibular symphysis (Figure 3). No mandibular drift or conformational changes to the temporomandibular joints had occurred, and the patient had a functional left mandible. The patient remained unneutered at time of the report as the priority was treatment of the oral cancer and developmental palatal defect, both of which were considered potentially life-threatening. The owners were advised of the possible genetic basis of the palatal defect. The patient has continued to do well 7 months after subtotal mandibulectomy, with a normal occlusion for the breed and no signs of oral pain or reoccurrence of the tumor.

## Discussion

This is the first report of spontaneous regeneration of the mandible following oral oncological surgery in a dog. Unlike older dogs that undergo partial mandibulectomy for tumor removal, this patient spontaneously regenerated the mandibular body within 8 weeks of the initial surgery. In humans, young patients who undergo mandibulectomy have been reported to regenerate new bone from progenitor cells within the remaining periosteum (13). For example, spontaneous regeneration has been documented in cases of *en bloc* excision of benign and malignant mandibular neoplasia, gunshot wounds, and other trauma in pediatric human patients (14). The degree of regeneration ranges from partial to complete regeneration of the mandible, taking anywhere from weeks to months before radiographic evidence of bone formation is present, and the majority of the described cases had preserved periosteum in the region (14). Appositional growth along periosteum is a main component to initial mandibular growth and development (15). Periosteum was preserved ventrally during this puppy’s surgery, and it was likely imperative to the subsequent regeneration of the bone. However, exact mechanisms by which bone was regenerated cannot be fully understood in this case.

Early detection of the oral tumor likely played an important role in achieving long term remission surgery. A subtotal mandibulectomy preserved adjacent periosteum, which may not have been possible if the tumor size and local invasion had made more extensive excision necessary. Anatomy such as the dentition and the neurovascular structures of the mandibular canal of the regenerated mandible cannot reform due to the loss of the necessary progenitor cells present during embryologic development. Nonetheless, regeneration of the body of the left mandible resulted in a functional lower jaw with no mandibular drift. Serial oral examinations following regrowth confirmed no signs of traumatizing malocclusion, and the patient continued to prehend food and water with ease.

Reconstructive surgery following segmental or partial mandibulectomies in dogs has been described to help address the functional disturbances associated with these surgeries, such as traumatic malocclusion (7, 8, 16). In young patients, reconstruction following excision would be complicated further by the need to account for continued growth (13). Current techniques use internal fixation of mandibular segments to facilitate the bone graft placement and regeneration, but this rigid fixation can inhibit natural growth in this area. Based on the spontaneous mandibular regeneration observed in this case, surgeons can consider monitoring for total or partial reformation of bone following mandibulectomy in skeletally immature dogs prior to pursuing reconstructive surgery. Notably, the likelihood of spontaneous regeneration is unpredictable at this time, as further studies would be needed to elucidate the incidence and extent of maxillofacial bone regeneration in immature dogs to help predict postoperative outcomes when presented with a similar case in the future.

## Data availability statement

The original contributions presented in the study are included in the article/supplementary material, further inquiries can be directed to the corresponding author.

## Ethics statement

Ethical review and approval was not required for the animal study because the case report is a description of a clinical case. Written informed consent was obtained from the owners for the participation of their animal in this study.

## Author contributions

AW: Writing – original draft. SP: Writing – review & editing. NF: Writing – review & editing.

## References

[1] Peralta S Webb SM Katt WP Grenier JK Duhamel GE. Highly recurrent BRAF p.V595E mutation in canine papillary oral squamous cell carcinoma. Vet Comp Oncol. (2023) 21:138–44. doi: 10.1111/vco.12869 36451536

[2] Nemec A Murphy BG Jordan RC Kass PH Verstraete FJM. Oral papillary squamous cell carcinoma in twelve dogs. J Comp Pathol. (2014) 150:155–61. doi: 10.1016/j.jcpa.2013.07.007, PMID: 24016780

[3] Ogilvie GK Sundberg JP O’Banion MK Badertscher RR Wheaton LG Reichmann ME. Papillary squamous cell carcinoma in three young dogs. J Am Vet Med Assoc. (1988) 192:933–6. PMID: 3366682

[4] Furuta K Nishi K Park CH Maeda K Iwai S Sakonjyu I. A case of papillary squamous cell carcinoma in the mandible of a young French bulldog. Can Vet J. (2021) 62:1181–4. PMID: 34728843 PMC8543691

[5] Soukup JW Snyder CJ Simmons BT Pinkerton ME Chun R. Clinical, histologic, and computed tomographic features of Oral papillary squamous cell carcinoma in dogs: 9 cases (2008–2011). J Vet Dent. (2013) 30:18–24. doi: 10.1177/089875641303000102, PMID: 23757821

[6] Umphlet R Johnson A Eurell JC Losonsky J. The effect of partial rostral Hemimandibulectomy on mandibular mobility and temporomandibular joint morphology in the dog. Vet Surg. (1988) 17:186–93. doi: 10.1111/j.1532-950X.1988.tb00996.x, PMID: 3238891

[7] Arzi B Verstraete FJM Garcia TC Lee M Kim SE Stover SM. Kinematic analysis of mandibular motion before and after mandibulectomy and mandibular reconstruction in dogs. Am J Vet Res. (2019) 80:637–45. doi: 10.2460/ajvr.80.7.637, PMID: 31246128

[8] Boudrieau RJ Mitchell SL Seeherman H. Mandibular reconstruction of a partial hemimandibulectomy in a dog with severe malocclusion. Vet Surg. (2004) 33:119–30. doi: 10.1111/j.1532-950X.2004.04019.x, PMID: 15027973

[9] Bar-Am Y Verstraete FJM. Elastic training for the prevention of mandibular drift following mandibulectomy in dogs: 18 cases (2005-2008). Vet Surg. (2010) 39:574–80. doi: 10.1111/j.1532-950X.2010.00703.x, PMID: 20459496

[10] Peralta S Fiani N Kan-Rohrer KH Verstraete FJM. Morphological evaluation of clefts of the lip, palate, or both in dogs. Am J Vet Res. (2017) 78:926–33. doi: 10.2460/ajvr.78.8.926 28738009

[11] Fiani N Peralta S. Extended subtotal mandibulectomy for the treatment of oral tumors invading the mandibular canal in dogs—a novel surgical technique. Front Vet Sci. (2019) 6:6. doi: 10.3389/fvets.2019.00339 31637248 PMC6787549

[12] Warshaw SL Carney PC Peralta S Fiani N. Piezosurgical bone-cutting technology reduces risk of maxillectomy and mandibulectomy complications in dogs. J Am Vet Med Assoc. (2023) 1–7:1–7. doi: 10.2460/javma.23.03.0130 37225159

[13] Shahzad F. Pediatric mandible reconstruction: controversies and considerations. Plast Reconstr Surg Glob Open. (2020) 8:e3285. doi: 10.1097/GOX.0000000000003285 33425597 PMC7787291

[14] Rai S Rattan V Jolly SS Sharma VK Mubashir MM. Spontaneous regeneration of bone in segmental mandibular defect. J Maxillofac Oral Surg. (2019) 18:224–8. doi: 10.1007/s12663-018-1153-9 30996542 PMC6441417

[15] Hennet PR Harvey CE. Craniofacial development and growth in the dog. J Vet Dent. (1992) 9:11–8. doi: 10.1177/089875649200900201 1290596

[16] Arzi B Verstraete FJM Huey DJ Cissell DD Athanasiou KA. Regenerating mandibular bone using rhBMP-2: part 1-immediate reconstruction of segmental Mandibulectomies. Vet Surg. (2015) 44:403–9. doi: 10.1111/j.1532-950X.2014.12123.x 24410740 PMC4451165

